# Impact of reimbursement systems on patient care – a systematic review of systematic reviews

**DOI:** 10.1186/s13561-024-00487-6

**Published:** 2024-03-16

**Authors:** Eva Wagenschieber, Dominik Blunck

**Affiliations:** Department of Healthcare Management, Institute of Management, Friedrich-Alexander-Universität Erlangen-Nürnberg, Lange Gasse 20, 90403 Nuremberg, Germany

**Keywords:** Reimbursement, Fee-for-service, Pay-for-performance, Bundled payment, Process, Structure, Outcome, Patient treatment, Systematic review

## Abstract

**Background:**

There is not yet sufficient scientific evidence to answer the question of the extent to which different reimbursement systems influence patient care and treatment quality. Due to the asymmetry of information between physicians, health insurers and patients, market-based mechanisms are necessary to ensure the best possible patient care. The aim of this study is to investigate how reimbursement systems influence multiple areas of patient care in form of structure, process and outcome indicators.

**Methods:**

For this purpose, a systematic literature review of systematic reviews is conducted in the databases PubMed, Web of Science and the Cochrane Library. The reimbursement systems of salary, bundled payment, fee-for-service and value-based reimbursement are examined. Patient care is divided according to the three dimensions of structure, process, and outcome and evaluated in eight subcategories.

**Results:**

A total of 34 reviews of 971 underlying primary studies are included in this article. International studies identified the greatest effects in categories resource utilization and quality/health outcomes. Pay-for-performance and bundled payments were the most commonly studied models. Among the systems examined, fee-for-service and value-based reimbursement systems have the most positive impact on patient care.

**Conclusion:**

Patient care can be influenced by the choice of reimbursement system. The factors for successful implementation need to be further explored in future research.

**Supplementary Information:**

The online version contains supplementary material available at 10.1186/s13561-024-00487-6.

## Background

The health care system has a variety of payment and reimbursement systems that provide different financial incentives for patient care. Every payment system carries incentives to over- or underprovide care. There is no optimal solution, as there is constant pressure to adapt and reform in order to ensure the best possible quality of care. Health care systems are reaching their financial limits and therefore it is desirable to achieve an increase in efficiency in patient treatment and, for example, to avoid unnecessary interventions [1]. To achieve this, health policy must ensure a regulatory framework in which health status is also an economic incentive for all actors in the health system, promoting health benefits and reducing economic disincentives.

Physicians have a stronger position in the physician–patient relationship because of the knowledge and information advantage, and problems arise in the provision of care when physicians’ financial interest do not match the patients’ need for treatment [2]. In addition to medical necessity, economic and financial factors also play a key role in patient treatment. Medical decisions in the inpatient sector are influenced daily by economic requirements, economic considerations, and financial resources, potentially with negative consequences for the quality of treatment and patient safety. In the hospital setting, economization is exemplified in that physicians often feel ethical conflicts and economic goals occur at the expense of adjustments in length of stay, case numbers, and patient selection [3]. The influence on patient care is examined under four different reimbursement systems: Salary, bundled payment, fee-for-service (FFS), value-based reimbursement. With a fixed salary, remuneration is based solely on the duration of working hours, whereas the type and volume of service, as well as the number of treatment cases or patients enrolled, have no influence on financial income. At the same time, both an advantage and a disadvantage in this reimbursement system is the dependence of the quality of treatment on the intrinsic motivation of the provider [2]. Bundled payment is the term for payments such as capitation or disease related groups (DRGs). Services are combined and “bundled” for payment during a single patient contact or over a temporal episode. One disadvantage of this reimbursement system is the incentive for health care providers to treat as many patients as possible with as little effort as possible and thus to engage in risk selection. On the other hand, this can increase the incentive for preventive measures on the part of health care providers [4]. In FFS reimbursement, the provider’s fee is based on the volume of services rendered. Shared-savings payment models are a mix of FFS and a fixed salary where providers participate from savings they achieve in patient care. This creates the disadvantage of FFS reimbursement that service providers will unnecessarily expand the number of services for monetary reasons, resulting in unnecessary care at the expense of payers and potentially patients. On the other hand, (potentially expensive) diseases can be identified and treated earlier through increased preventive measures [2, 5]. Value-based reimbursement additionally promotes the quality and success of medical procedures. Remuneration is expanded to the extent that it is linked to predefined quality targets at the levels of transparency, accessibility to care, indication, structure, process or outcome. While value-based reimbursement can promote the intrinsic motivation of providers, care must be taken to ensure that there is no risk selection for patients who can be treated well or that there are no negative spill-over effects into other areas of treatment. Another disadvantage of this reimbursement system is the large number of factors besides medical treatment that contribute to recovery, such as comorbidities or socioeconomic factors [1].

### Aim

Other reviews have addressed effects on patient care in outpatient settings [6] or included studies from developing countries in their evaluations [7]. Previous studies only focus on specific areas of patient care [8], are not methodologically designed as a systematic review [9], focus only on individual specialties [10] or reimbursement systems [11] and do not compare the effect of different reimbursement systems. A comprehensive and structured overview, comparing the outcomes of several reimbursement systems on areas of patient care, is missing.

The objective of this paper, thus, is to provide a review of systematic reviews on the relationship between reimbursement systems and patient care. The research question is narrowed down using the PICOS algorithm: Physicians (Population), Reimbursement systems (Intervention), different reimbursement systems or differences over time (Comparison), effects on patient care divided into the parameters structure, process, outcome (Outcome), systematic reviews and meta-analyses (Study type). The aim is to analyze how reimbursement systems affect patient care across countries.

## Materials and methods

The systematic review follows the guidelines of the PRISMA (Preferred Reporting Items for Systematic Reviews and Metaanalyses) statement [12], has been performed via the databases PubMed, Web of Science and Cochrane Database of Systematic Reviews between 02/12/2021 and 22/12/2021 and has been complemented with an additional search on Google Scholar and in the reference lists of relevant studies. The search term was formed by linking keywords and their synonyms from previously published relevant studies on the three aspects of the research questions: impact, reimbursement systems, and patient care (see Table 1 for the full search term for each database).

**Table 1:** Search terms

**Database**	**PubMed**
**Date of Search**	02.12.2021
**Search Strategy**	(incentiv* [Title/Abstract] OR "financial incentiv*" [Title/Abstract] OR effect* [Title/Abstract] OR impact* [Title/Abstract] OR influence* [Title/Abstract]) AND (reimburs* [Title/Abstract] OR "reimbursement system" [Title/Abstract] OR "reimbursement mechanism" [Title/Abstract] OR "pay for performance" [Title/Abstract] OR "pay-for- performance"[Title/Abstract] OR p4p[Title/Abstract] OR "fee for service" [Title/Abstract] OR "fee-for-service"[Title/Abstract] OR FFS[Title/Abstract] OR capitat* [Title/Abstract] OR "value based reimbursement" [Title/Abstract] OR "value-based reimbursement"[Title/Abstract] OR salar* [Title/Abstract] OR "payment system*" [Title/Abstract]) AND ("health care" [Title/Abstract] OR "quality of health care" [Title/Abstract] OR "patient care" [Title/Abstract] OR "medical care" [Title/Abstract] OR "medical treatment" [Title/Abstract] OR quality* [Title/Abstract] OR effectiveness* [Title/Abstract] OR productiv* [Title/Abstract] OR performance* [Title/Abstract] OR behavior* [Title/Abstract] OR behaviour* [Title/Abstract] OR outcome*[Title/Abstract])
**Results**	312
**Filter**	Time interval: 2011 – 2021 Article type: Systematic Reviews, Meta-Analysis A language filter was not applied to identify all potentially relevant studies
**Database**	**Web of Science**
**Date of Search**	02.12.2021
**Search Strategy**	(AB = (incentiv*) OR AB = ("financial incentiv*”) OR AB = (effect*) OR AB = (impact*) OR AB = (influence*)) AND (AB = (reimburs*) OR AB = ("reimbursement system") OR AB = ("reimbursement mechanism") OR AB = ("pay for performance") OR AB = ("pay-for-performance") OR AB = (p4p) OR AB = ("fee for service") OR AB = ("fee-for-service") OR AB = (FFS) OR AB = (capitat*) OR AB = ("value based reimbursement") OR AB = ("value-based reimbursement") OR AB = (salar*) OR AB = (payment system*)) AND (AB = ("health care") OR AB = ("quality of health care") OR AB = ("patient care") OR AB = ("medical care") OR AB = ("medical treatment") OR AB = (quality*) OR AB = (effectiveness*) OR AB = (productiv*) OR AB = (performance*) OR AB = (behavior*) OR AB = (behaviour*) OR AB = (outcome*))
**Results**	869
**Filter**	Time interval: 2011–2021 Article type: Review A language filter was not applied to identify all potentially relevant studies
**Database**	**Cochrane Database of Systematic Reviews**
**Date of Search**	02.12.2021
**Search Strategy**	incentiv* OR "financial incentiv*" OR effect* OR impact* OR influence*AND reimburs* OR "reimbursement system" OR "reimbursement mechanism" OR "pay for performance" OR "pay-for- performance" OR p4p OR "fee for service" OR "fee-for-service" OR FFS OR capitat* OR "value based reimbursement" OR "value-based reimbursement" OR salar* OR "payment system*"AND "health care" OR "quality of health care" OR "patient care" OR "medical care" OR "medical treatment" OR quality* OR outcome* OR effectiveness* OR productiv* OR performance* OR behavior* OR behaviour*
**Results**	36
**Filter**	Time interval: 2011–2021 Fields: Title/Abstract/Keywords A language filter was not applied to identify all potentially relevant studies

Inclusion criteria are defined as (a) the paper must be a systematic review or meta-analysis, (b) the countries considered must be industrialized nations, and (c) the effect of payment/reimbursement systems on patient care was examined.

The search period is set to ten years and only studies published in German or English were included. All records were exported to EndNote 20 [13] and screened by the authors; disagreements were solved by discussion. All studies categorized as “relevant” or “uncertain” in this step were analyzed in full text.

Studies categorized as relevant after full text analysis were included in this work and assessed for study quality using the AMSTAR-2 score, which is a comprehensive questionnaire to assess systematic reviews of (non)randomized trials [14]. Using the framework of Donabedian, the results are divided into the three dimensions structure, process, outcome [15] (see Table 2). The structure dimension combines the following parameters: “unintended consequences” and “organizational changes”. Unintended consequences are mostly related to changes in risk selection or spill-over effects, whereas organizational changes are related to effects in personnel structures, for example. The dimension of structure is of particular interest for health care authorities as well as payers as it shapes the organizational characteristics of how care is delivered.

**Table 2:** Dimensions and outcome categories based on the Donabedian model for quality of care

Dimension	Donabedian framework	Category	Exemplary content
Structure	Physical and organizational characteristics where healthcare occurs	Unintended consequences	Risk selection, spill-over effects
		Organizational changes	Effects on personnel structure
Process	Focus on the care delivered to patients e.g. services, diagnostics or treatments	Resource utilization	Readmission rates, length of stay
		Access	Socioeconomic inequalities in the utilization of health care services
		Behavior	Intrinsic motivation, documentation
Outcome	Effect of healthcare on the status of patients and populations	Quality/Health outcomes	Mortality, treatment quality
		Efficiency	Effects on direct savings for medical services
		Economic effects	Effects on total social expenditures

The categories “resource utilization”, “access”, and “behavior” are combined under the parameter process. While resource utilization mostly describes changes in readmission rates or length of stay, the access category reflects socioeconomic inequalities in the utilization of health care services. The behavior category includes effects related to intrinsic motivation, preventive services provided by physicians, or documentation of health parameters, among others. The dimension of process defines how providers deliver care as well as the points of contacts for patients.

The outcome dimension, on the other hand, combines the parameters “quality/health outcomes”, “efficiency”, and “economic effects”. Actual changes in mortality, treatment quality, screening or vaccination rates are mapped in the “quality/health outcomes” category. The “efficiency” category deals with the effects on direct savings in the provision of a specific medical service or effects on salaries, whereas the “economic effects” category records effects that are significant for society. The dimension of outcome could be regarded of the main value driver from a patient perspective as it answers to what extent patients’ original need for care is fulfilled. Furthermore, outcomes are of particular interest for payers, as payers commonly decide, for example, what services are reimbursed and therefore potentially have a high interest in a positive cost-outcome-relation.

For all reimbursement systems described, the number of included studies, as well as the examined medical specialties or physician groups and countries in which the interventions are carried out, are also transferred in each case. For each reimbursement system described, it is examined whether it improved or worsened the outcome categories of patient care, whether there were heterogeneous results, or whether no difference was found in the outcome categories before and after the intervention. The frequency reviews found an improvement, worsening, heterogeneous outcome, or no difference for each payment system per outcome category were summarized in a single table. In this study, increases in healthcare utilization, documentation of health parameters, and higher screening rates or lower mortality rates are defined as improvements. A measurable increase in risk selection, negative spill-over effects, longer hospital stays, or higher readmission rates are considered deteriorations in patient care. In the economic categories of efficiency and economic effects, savings in health care spending and total societal spending, respectively, are considered as improvements. Reviews finding heterogeneous results include studies with conflicting findings, because some of the included primary studies find positive results in one category, whereas other primary studies find negative effects or no significant effects at all, leaving the study or respective review with an overall heterogeneous result. It is assumed that health care is optimized by an increase in health care services, shorter lengths of stay, more efficient care, and lower overall societal health care expenditures.

## Results

### Overview

**Fig. 1:**
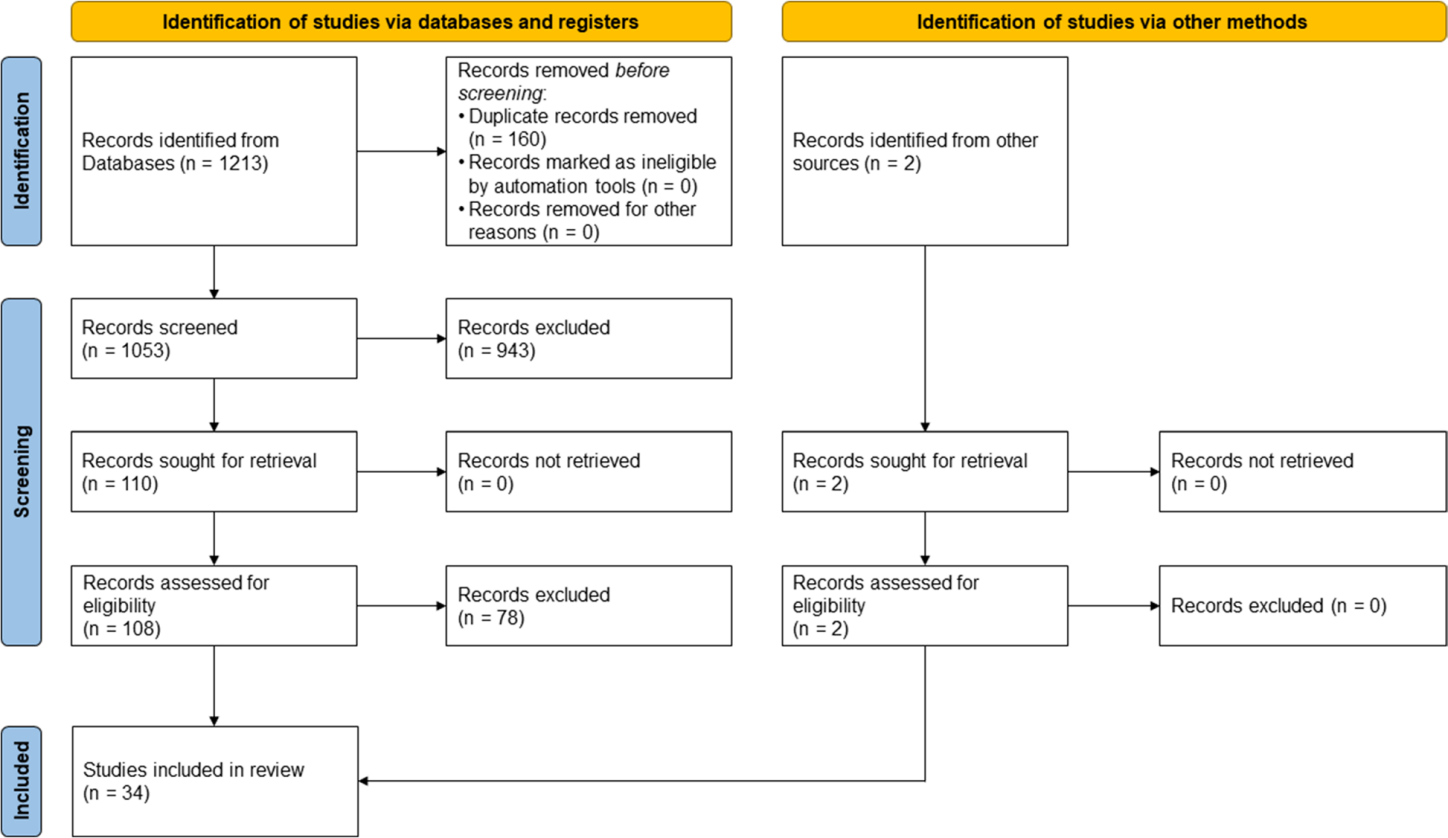
Flowchart

A total of 1,213 hits were identified by the database search on 02/12/2021, with 2 additional hits identified by the search in Google Scholar. After duplicates were removed, 1,053 abstracts were screened by both authors, resulting in 943 hits being initially excluded. The remaining 110 hits were analyzed in full text, whereupon 34 hits were included in this work (see Fig. 1).

Overall, the 34 included systematic reviews describe the influences on patient care based on a total of 971 primary studies. Ten of the 34 included reviews are rated as high quality, 16 as moderate quality, and eight as low quality according to the assessment procedure using the AMSTAR-2 questionnaire (see Table 3). Some of the identified systematic reviews examined more than one reimbursement system. Therefore, for the sake of clarity, we refer to a total number of 60 studies in the following. Of these, the reimbursement system salary was investigated in four studies, bundled payments in 15, FFS payments in a further eleven studies and value-based reimbursement in a total of 29 studies. Out of the 60 studies 45 were conducted in the USA, 38 in European countries, 28 in the UK, 23 in other countries and 17 in Canada. An overview of the results is provided in Table 4.. In the following, we describe the results of the systematic review regarding Donabedian’s categories of quality: structure, process, and outcome.

**Table 3:** Quality rating

Review	AMSTAR-2 Score	Quality rating	Risk of bias rating	Reimbursement system
Agarwal et al.	7 of 13	moderate	moderate	BP^a^
Ahmed et al.	4 of 13	low	high	VBP^b^
Barouni et al.	5 of 13	low	high	BP
Benabbas et al.	7 of 13	moderate	moderate	VBP
Bernstein et al.	10 of 13	high	low	BP
Brocklehurst et al.	10 of 13	high	low	BP, FFS^c^
Brown et al.	8 of 13	moderate	moderate	BP, FFS, VBP
Carter et al.	9 of 13	high	low	BP, FFS, VBP
Cattel et al.	6 of 13	moderate	moderate	BP
de Bruin et al.	3 of 13	low	high	VBP
de Vries et al.	7 of 13	moderate	moderate	BP, FFS, VBP
Eijkenaar et al.	7 of 13	moderate	moderate	VBP
Emmert et al.	7 of 13	moderate	moderate	VBP
Feldhaus et al.	3 of 13	low	high	BP, FFS, VBP
Forbes et al.	10 of 13	high	low	VBP
Gillam et al.	4 of 13	low	high	VBP
Gupta et al.	12 of 13	high	low	VBP
Heider et al.	8 of 13	moderate	moderate	Salary, BP, FFS, VBP
Herbst et al.	4 of 13	low	high	VBP
Huang et al.	12 of 16	high	low	VBP
Jia et al.	14 of 16	high	low	FFS, VBP
Kim et al.	8 of 13	moderate	moderate	VBP
Kondo et al.	7 of 13	moderate	moderate	VBP
Langdown et al.	4 of 13	low	high	VBP
Lee et al.	6 of 13	moderate	moderate	VBP
Markovitz et al.	3 of 13	low	high	VBP
Martin et al.	6 of 13	moderate	moderate	VBP
Mathes et al.	12 of 13	high	low	VBP
Mauro et al.	8 of 13	moderate	moderate	VBP
Mendelson et al.	9 of 13	high	low	VBP
Mitchell et al.	8 of 13	moderate	moderate	VBP
Palmer et al.	10 of 16	moderate	moderate	BP
Quinn et al.	10 of 13	high	low	Salary, BP, FFS
Vlaanderen et al.	7 of 13	moderate	moderate	VBP

^a^*BP* Bundled Payment

^b^*VBP* Value-based payment

^c^*FFS*: Fee-for-service

### Structure

**Table 4.**
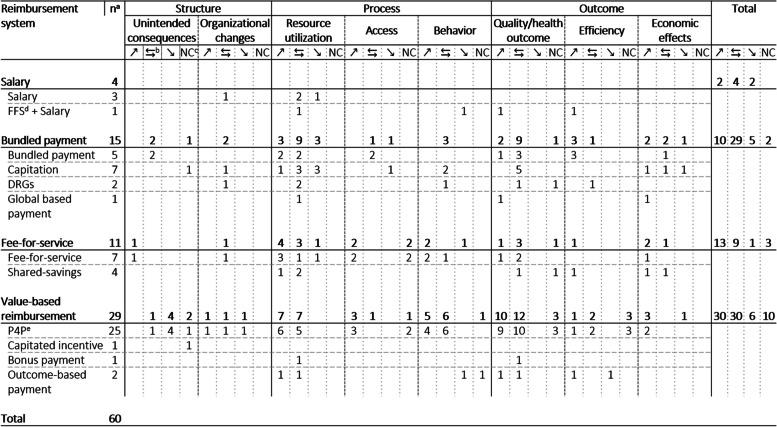
Reported effects on quality of care category for different reimbursement systems

^a^*n* Number of studies. ^b^⇆: heterogeneous. ^c^*NC*: No change. ^d^*FFS*: Fee-for-service. ^e^*P4P*: Pay-for-performance

#### Unintended consequences

No unintended consequences in patient care are found for the salary payment system. Studies find heterogeneous results for this category for bundled payments in form of a decrease in treatment volume while there is an increase in risk selection and case complexity [16, 17]. An association was found between bundled payments and patient selection based on sociodemographic factors and comorbidities [16]. Positive changes were noted in indicators that were not included in the FFS model; these were, however, only short-term [18]. Some reviews find unintended changes after implementation of pay-for-performance models (P4P), a type of value-based reimbursement, in form of risk selection, spill-over effects, protocol-driven and less patient-centered care and neglect of non-incentive indicators [19–23]. Some studies find no evidence for a change in patient risk selection in their included primary studies [24, 25].

#### Organizational changes

There are heterogeneous results on the impact on patient care after the introduction of different payment systems. One study reports effects in the form of increasing numbers of physicians per patient and decreasing numbers for bundled payments [26]. While one review finds heterogeneous results for salary, bundled payment, FFS, and value-based payment for the structural organization of patient care [27], others find both positive and negative effects for value-based payment as an improvement in care management processes or a worse organization of large hospitals [28, 29].

### Process

#### Resource utilization

Reviews find heterogeneous effects for salary models differentiated by specialty. While induction time and total treatment time increase in anesthesiology, outpatient visits and surgical procedures decrease in gynecology [30]. When salary and FFS payments are combined, a decrease in clinical services per year and in hospital readmissions is noted [27, 30]. Within models of bundled payments, heterogeneous results are found: While one source describes a decline in all-cause hospitalizations and readmissions [30], other sources find both improvements and deterioration in hospital facility use and the number of acute admissions [27, 31]. Deteriorations are described in the following categories: use of patient care resources, number of services provided per patient, shorter lengths of stay, discharges to post-hospital facilities [16, 18, 24, 30, 32]. Some reviews find both differences and no differences in the use of health care resources after the introduction of bundled payments [17, 27, 30]. Within DRG models, evidence is heterogeneous and describes no change, an increase, or a decrease in hospital readmissions and in the length of stay [26, 33]. For global-based payment, evidence is heterogeneous in terms of higher or lower utilization, and no change in resource utilization [34]. The heterogeneity of influences on health care resource utilization continues for FFS payments as sources find an increase in the number of physician visits per patient [18, 24], a reduction in length of stay and computer tomography exams [30–32] or heterogeneous results for process indicators [27]. Negative effects include an increase in the number of patients per physician [35]. In P4P models, six studies report an improvement in resource utilization as an increase in health care services, physician visits and a shorter length of stay [20, 25, 28, 32, 36, 37]. Other reviews come to very heterogeneous results regarding the change in resource utilization after the introduction of P4P models in the following categories: health care and resource utilization, length of stay, readmission rates, process indicators [10, 11, 27, 38].

#### Access

There is no research showing an impact of salary on access to health care. Bundled payments show heterogeneous results in form of changes of the patient structure with respect to insured status or a decline in patients with home dialysis [17, 30]. Studies examining FFS payment may also measure the impact on access to care. Improvements are noted in waiting time and a reduction of patients who leave the health care provider without treatment [30, 35]. No differences were found in the treatment of social or ethnic inequalities [18, 24]. For value-based models, results are heterogeneous regarding the impact on access to patient care. Among them, three studies identify a positive impact after the introduction of P4P models in form of an increase in equity of access to care and a decrease in social inequalities [20, 32, 36]. Other results show no significant reduction in access for disadvantaged groups or no improved access to primary care [11, 19].

#### Behavior

Salary models lead to a decrease in hours worked per week [30]. For bundled payment models, the results show both increases and decreases, means heterogeneous results, in the number of preventive consultations [18] and services as well as increases in preventive consultations, reported illness severity and referral to post-acute care facilities after hospitalization [24, 33]. An increased number of services provided were reported for FFS models [18, 24, 35]. Positive changes after the introduction of P4P models were noticed in some categories: increased use of computers and documentation of care, diabetes tests, physician behavior [11, 20, 35, 39]. Other reviews find results that are more heterogeneous on effects on the behavior in patient care [10, 22, 23, 25, 36, 40]. For example, an improved data collection leads to increased pressure on physicians and thereby provoke negative behavior change [36]. General heterogeneous effects in terms of a disruption of patient-centered care with less focus on patient needs are reported as well as an increase in blood pressure checks and an improvement in intrinsic motivation among care providers [10, 23, 25, 40]. Both, an increase and no change in medication prescription is found in two value-based models [10, 41].

### Outcome

#### Quality/health

One review finds a decrease in transfer rates out of hospitals for a salary-based payment [30]. The results for bundled payments are heterogeneous [18, 27, 30, 31]. Heterogeneous results, which means improvements as well as decreases and no changes are found within the primary studies in the reviews for mortality, rehospitalization rates, quality of care and numbers of treatment cases [16, 27, 30, 31, 42]. Some reviews notice an improvement in the quality and number of screenings [30, 42] or a decrease in the case complexity [16]. Evidence of the impact on quality of care and health outcomes associated with P4P is also examined in reviews. One review reports improvement in terms of an increase in immunization rates among children for FFS payments [35], whereas other sources find increases, decreases and no changes in number of treatment cases, treatment outcomes, mortality, and hospitalization rates [18, 27, 31]. The most influences on health outcomes or quality of care are found in models of value-based payment. Nine reviews find evidence of improvement with P4P models in these categories: immunization rates [35, 43], specific clinical values (e.g., cholesterol, blood pressure, screening rates, birth weight) [21, 39, 42, 44], quality of care [23, 28, 45]. Heterogeneous outcomes are found in another ten reviews [11, 19, 20, 22, 27, 36, 38, 40, 46, 47]. Among these, positive as well as negative results are found in patient-related health outcomes [19, 27], complication rates [38], health outcomes, quality of care and screening rates [22, 47]. Other sources report heterogeneous effects in patient satisfaction, short-term health outcomes and mortality [20, 22, 40, 47]. No effects on mortality, quality of care, health outcomes, rehospitalization or patient satisfaction after an implementation of value-based reimbursement are described in six reviews [11, 20, 31, 37, 38, 46].

#### Efficiency

When providers are reimbursed with fixed salaries in combination with FFS elements, the annual salary increases [30]. Bundled payments have a positive impact on the efficiency in terms of a decrease in health care spending and hospitalizations [16, 30, 42]. Furthermore, heterogeneous results, means deterioration as well as improvement, in treatment costs are described in one review [26]. Shared-savings models were found to lead to a reduction in perinatal care spending [42]. An improvement in the cost-effectiveness of treatments in P4P models by reducing costs was found in one review [19]. Other sources present heterogeneous results in terms of both positive and negative effects on the (marginal) costs of care [29, 38]. No evidence for changes in efficiency are determined in three other reviews [20, 22, 45].

#### Economic effects

For bundled payments, the results are very heterogeneous. Cuts in health spending as well as increases, no changes or unclear effects are noted [31, 32, 34]. When payment is based on FFS models, positive effects on health care spending are most often found [18, 32]. One study, however, reports heterogeneous effects [31]. The results on the impact of value-based payment models on economic conditions are mostly positive, as they lead to a reduction in the growth of health care spending and costs [32, 41, 44].

## Discussion

### Principal results

To answer the question of the relationship of different reimbursement systems and patient care, we conducted a systematic review of systematic reviews in order to structure the existing body of evidence in this topic. We identified 34 studies analyzing 60 reimbursement systems and structured the results from the perspective of the Donabedian framework.

For the reimbursement of health care providers via salary, the results show little to no influence on the subcategories of the dimension structure. For the dimension process, the results are heterogeneous with a tendency toward deterioration, manifested in a reduction in services rendered and hours worked. The classic disincentives of salary-based reimbursement, minimization of the quantity of services and treatments, are confirmed in the results. The categories of the outcome dimension, on the other hand, are clearly improved, with a decrease in hospital discharge rates and an increase in income. The certainty of these results is high due to the high study quality and the risk of bias is low, since three high-quality studies and one medium-quality study were included in the evaluation.

The studies on bundled payments show few and heterogeneous effects on the structural dimension of patient care. The resource utilization subcategory shows heterogeneous results, with most results being equally positive and negative. The remaining categories in the process dimension appear to have mostly heterogeneous effects. Overall, bundled payments are found to have more positive effects on patient care in the outcome dimension categories. The disincentives of bundled payments are confirmed in the form of reductions in services, but also refuted in the form of shorter lengths of stay and lower readmission rates in hospitals. When interpreting the results, the rather below-average study quality must be considered. Although five high-quality reviews examine the effects of the bundled payments, eight reviews with a medium quality and four papers with a low quality are also included in the evaluations, so that the certainty of results is limited and there is a risk of bias.

In the results for FFS models, especially the categories in the dimension process tend to be positively affected. While access to health care and provider behavior tend to be mostly positive, there are as many heterogeneous and negative effects for resource utilization as positive ones. Measured health impact is very heterogeneous and tend to be negative, while efficiency and economic impacts tend to be improved. An increase in the number of health care services, a classic disincentive, is directly confirmed by several studies. The quality of the included reviews and, thus, also the certainty of results tends to be high, since seven reviews with a low risk of bias, four with a medium and only one review with a high risk of bias are included in the evaluation.

For models of value-based reimbursement, results are inconclusive or more negative with respect to subcategories of the structural dimension, noting changes in risk selection, negative spillover effects, and a shift away from patient-centered care [19–23]. In contrast, these payment models achieve substantial improvements in the process dimension and specifically in resource utilization. Although the effects on health outcomes are heterogeneous for P4P models, they indicate a clear tendency toward improvement, whereas no clear improvements or deteriorations were found for the other two subcategories. The misaligned incentives of value-based payment in the form of patient selection described at the beginning are both confirmed [21–23] and refuted [24, 25]. The quality of the included reviews and thus also the certainty of results is average overall. Although seven of the relevant reviews are of high quality, 15 have a medium risk and seven have a high risk of bias, which may affect the results.

Overall, the rate of identified improvements for FFS and VBP is the best compared to heterogeneous effects, deteriorations, or no identified changes. While about 50% of all identified results for FFS show improvements, it is 40% for VBP. On the other hand, only 25% of the identified outcomes for a salary are improvements and 21% for bundled payment. Across all reimbursement systems, most of the results were identified in the categories resource utilization and quality/health outcome. Especially the categories of the process and outcome dimension, specifically the subcategories resource utilization and health outcome are influenced by the choice of reimbursement models and cause a change in patient care. These categories therefore have a greater impact on the overall results than categories in which fewer results have been identified. Mainly models of bundled and value-based reimbursement are affected. The effects of FFS and value-based reimbursement are mostly positive in the results compared to the other two reimbursement systems. Both payment models tend to show positive effects in the categories of the process and outcome dimension, and cite an increase in health care services provided, a reduction in length of stay, an increase in screening rates of patients, and an improvement in health parameters. In the case of value-based reimbursement, however, many endpoints were found to have no or very heterogeneous effects following the introduction of these reimbursement models. Primarily, these endpoints are unintended consequences, resource use, behavior, health outcomes, and efficiency. Bundled payment models show more heterogeneous and more negative than positive results. These are found predominantly in the resource utilization and health outcome categories, indicating a more positive impact of FFS and value-based compensation. Salary receives heterogeneous results, with categories in the process dimension tending to worsen and those in the outcome dimension tending to improve. Although the disincentives of the respective reimbursement systems are confirmed for all models, refutations are found for bundled and value-based reimbursement regarding length of stay, readmission rates, negative spill-over effects and patient selection.

### Implication

In particular, the categories of the process and outcome dimension, more precisely defined as the subcategories resource utilization and quality/health outcome, are reported to be influenced by the choice of reimbursement model and cause a change in patient care. Models of bundled and value-based reimbursement seem to be particularly affected. The effects are more positive for FFS and value-based reimbursement in comparison to both other reimbursement systems. FFS as well as VBP models show positive effects in the process and outcome dimension categories, frequently citing an increase in health care services provided, a reduction in length of stay, an increase in patient screening rates, and an improvement in health parameters. Judging by the results and comparison of the four reimbursement systems, it is therefore worthwhile to further expand models of FFS and value-based reimbursement in the health care system and to investigate their successful implementation as well as potential moderating factors.

### Limitations

There are some limitations in this review. The AMSTAR-2 tool is only partly appropriate to evaluate the reviews because it also evaluates clinical studies and therefore might underestimate the actual quality of some reviews involved. Not all of the included reviews provide a clear definition of their view on improvement or deterioration of care. Individual primary studies may be integrated into the results of several studies of included reviews and have a greater influence on the analysis than other primary studies included in only one review which bears the risk of overestimation of certain results. When interpreting the results, it is important to note that FFS or P4P models cannot be applied to any health care system; rather, the exact conditions for successful implementation must be individually and critically examined. Finally, publication bias is a limitation and can lead to overrepresentation of improvements due to the implementation of the described reimbursement models. Future studies should also identify more relevant databases to increase the quality of the systematic review and the validity of the results. Additionally, future studies should analyze the monetarization of the effects and aim for a better comparability of study settings as difficulties arise from interpreting health policy analyses which were conducted in different settings as well as causal interpretation might be limited as most underlying studies were not conducted as randomized controlled trials.

### Supplementary Information

**Supplementary material 1. **

## Data Availability

No new data generated/Not applicable.

## Abbreviations

FFS: Fee-for-service
P4P: Pay-for-performance
